# The effects of 8 weeks of heavy resistance training and branched-chain amino acid supplementation on body composition and muscle performance

**DOI:** 10.1186/1550-2783-10-S1-P25

**Published:** 2013-12-06

**Authors:** Mike Spillane, Christamarie Emerson, Darryn S Willoughby

**Affiliations:** 1 Exercise and Biochemical Nutrition Lab, Department of Health, Human Performance, and Recreation, Baylor University, Waco, TX 76798, USA

## Purpose

This study determined the effects of eight weeks of heavy resistance training combined with branched-chain amino acid (BCAA) supplementation on body composition and muscle performance.

## Methods

Nineteen non-resistance-trained males resistance-trained (3 sets of 8-10 repetitions) four times/week for eight weeks while also ingesting 9 g/day of BCAA or 9 g/day of placebo (PLAC) on exercise days only (half of total dose 30 min before and after exercise). Data were analyzed with separate 2 x 2 ANOVA (p < 0.05).

## Results

For total body mass, neither group significantly increased with training (p = 0.593), and there also were no significant changes in total body water (p = 0.517). Also, no training- or supplement-induced (p = 0.783) changes occurred with fat mass or fat-free mass (p = 0.907). Upper-body (p = 0.047) and lower-body strength (p = 0.044) and upper- (p = 0.001) and lower-body muscle endurance (p = 0.013) were increased with training; however, these increases were not different between groups (p > 0.05).

## Conclusion

When combined with heavy resistance training for eight weeks, 9 g/day of BCAA supplementation, half given 30 min before and after exercise, had no preferential effects on body composition and muscle performance.

